# Inflammatory biomarkers and 30-day thoracic outcomes after surgical versus non-surgical management of spontaneous pneumothorax: a retrospective cohort study

**DOI:** 10.3389/fmed.2026.1868899

**Published:** 2026-07-03

**Authors:** Qingguo Ding, Yingding Ruan, Chuan Long, Wenjun Cao, Aiming Yang, Peng Sun, Jianwei Han

**Affiliations:** 1 Department of Thoracic Surgery, First People’s Hospital of Jiande, Jiande, China; 2 Department of Thoracic Surgery, Affiliated Zhongshan Hospital of Dalian University, Dalian, China; 3 Department of Radiology, First People’s Hospital of Jiande, Jiande, China

**Keywords:** 30-day thoracic outcomes, inflammatory biomarkers, inverse probability of treatment weighting, pneumothorax, surgical intervention

## Abstract

**Background:**

Systemic inflammatory biomarkers may reflect early recovery after spontaneous pneumothorax, but their association with short-term outcomes after surgical versus non-surgical management remains uncertain. This study evaluated inflammatory indices and 30-day thoracic outcomes after different treatment strategies.

**Methods:**

We retrospectively analyzed 320 eligible spontaneous pneumothorax episodes treated between January 2017 and December 2024, including 120 managed with video-assisted thoracoscopic surgery and 200 managed without surgery. The primary analysis was episode-based, with a patient-level sensitivity analysis retaining the first eligible episode per patient. The primary outcome was any thoracic complication within 30 days after discharge, and the secondary outcome was hospital stay after the index procedure. Stabilized inverse probability of treatment weighting was used to adjust baseline differences. Treatment-outcome associations were estimated using weighted regression models.

**Results:**

After weighting, video-assisted thoracoscopic surgery was associated with higher post-treatment inflammatory indices, including SII, PLR, NLR, and WBC, but lower LMR, albumin, and hemoglobin. Compared with non-surgical management, video-assisted thoracoscopic surgery was associated with fewer 30-day post-discharge thoracic complications (13.2% vs. 28.5%, *p* < 0.001), but longer hospital stay after the index procedure (median 5.00 vs. 3.00 days, *p* < 0.001) and more frequent antibiotic use (26.2% vs. 7.7%, *p* < 0.001). In weighted models, surgery remained associated with a lower risk of 30-day post-discharge thoracic complications (OR = 0.38, 95% CI = 0.21–0.71, *p* = 0.002) and longer hospital stay after the index procedure (*β* = 1.91 days, 95% CI = 0.34–3.49, *p* = 0.017).

**Conclusion:**

Video-assisted thoracoscopic surgery was associated with fewer 30-day post-discharge thoracic complications but longer hospitalization in spontaneous pneumothorax. Elevated post-treatment inflammatory biomarkers after surgery may reflect surgical stress rather than infection alone and should be interpreted cautiously.

## Introduction

Spontaneous pneumothorax (SP) is classically categorized as primary or secondary, depending on the presence of pre-existing pulmonary disease. Primary spontaneous pneumothorax (PSP) typically occurs in adult patients without overt pulmonary disease, whereas secondary SP is more common in patients with underlying conditions, including chronic obstructive pulmonary disease (COPD), emphysema, or tuberculosis (1, 2). Epidemiological studies indicate that PSP disproportionately affects men, accounting for approximately 73% of cases (2). Although PSP is formally defined as pneumothorax “without apparent lung disease,” high-resolution computed tomography (CT) studies demonstrate that 63–80% of patients with PSP present apical subpleural blebs or emphysematous changes (3, 4). However, the causal relationship between these radiological abnormalities and pneumothorax pathogenesis remains controversial, as emerging evidence suggests that diffuse pleural porosity and underlying inflammatory or genetic processes may represent more fundamental drivers than isolated bullae (5, 6). These observations highlight that SP is not only an anatomical air-leak disorder but may also involve broader structural and inflammatory mechanisms.

SP can be managed using conservative observation, oxygen therapy, needle aspiration, chest tube drainage, and surgery. In routine practice, however, patients selected for surgery often differ from those managed without surgery, particularly with respect to recurrence history, persistent air leak, radiological bullae, pneumothorax burden, or perceived risk of treatment failure. These differences complicate the interpretation of short-term outcomes after surgical versus non-surgical management in retrospective cohorts. Therefore, outcome comparisons require careful adjustment for baseline treatment-selection factors rather than simple unadjusted group comparisons.

Recent studies have increasingly focused on the use of inflammatory biomarkers as prognostic indicators in thoracic medicine. Substantial evidence has demonstrated that the neutrophil-to-lymphocyte ratio (NLR), platelet-to-lymphocyte ratio (PLR), lymphocyte-to-monocyte ratio (LMR), and systemic immune-inflammation index (SII) reflect systemic inflammatory status and immune homeostasis (7–9). The prognostic value of these biomarkers has been validated in acute myocardial infarction (7), COPD and idiopathic pulmonary fibrosis (10–12), and thoracic oncology (9, 13, 14). In lung cancer surgery, perioperative NLR, PLR, and SII are established predictors of postoperative pneumonia, atrial fibrillation, and survival (8, 15). Nevertheless, their predictive value in pneumothorax remains unclear, especially because post-treatment inflammatory indices may reflect both disease activity and treatment-related stress. In surgically treated patients, these biomarkers may be influenced by operative trauma and perioperative inflammation, rather than infection or pneumothorax severity alone.

The knowledge gap addressed in this study is therefore twofold: whether surgical management is associated with better 30-day thoracic outcomes after accounting for baseline differences between treatment groups, and how post-treatment inflammatory biomarkers should be interpreted in this clinical setting. Therefore, this study evaluated the association of treatment strategy and post-treatment inflammatory biomarkers with 30-day thoracic outcomes in patients with SP. Because treatment allocation was not random, stabilized inverse probability of treatment weighting (IPTW) was used to improve baseline comparability between the surgical and non-surgical groups. In addition, because recurrent episodes may introduce within-patient correlation and treatment-selection bias, a patient-level sensitivity analysis restricted to the first eligible episode was performed.

## Patients and methods

### Study population and eligibility criteria

This retrospective cohort study screened all patients with radiologically confirmed pneumothorax treated in the Department of Thoracic Surgery at the First People’s Hospital of Jiande between January 2017 and December 2024. The analytic cohort was restricted to eligible episodes of spontaneous pneumothorax, including primary and secondary spontaneous pneumothorax. Iatrogenic, traumatic, postoperative, malignant, acupuncture-related, and other non-spontaneous pneumothorax episodes were excluded.

Inclusion criteria were as follows: (1) radiologically confirmed pneumothorax on chest radiography or chest CT; (2) spontaneous onset without traumatic, iatrogenic, postoperative, or malignant cause; (3) age ≥15 years; (4) treatment with either chest drainage alone or chest drainage followed by VATS during the index episode; and (5) available clinical, laboratory, and follow-up data for outcome assessment.

Exclusion criteria were: (1) traumatic, iatrogenic, postoperative, malignant, or acupuncture-related pneumothorax; (2) pneumothorax associated with active pulmonary infection at admission; (3) autoimmune disease requiring systemic glucocorticoid or immunosuppressive therapy; (4) pregnancy or incarceration; (5) consecutive surgery within 30 days for another condition; (6) readmission solely for insurance reimbursement or administrative reasons without a new clinical event; and (7) incomplete clinical, laboratory, imaging, or follow-up data.

This study complied with the Declaration of Helsinki and was approved by the Ethics Committee of the First People’s Hospital of Jiande (Approval Number: 20250930-KY-02-001). The requirement for informed consent was waived because of the retrospective nature of the study.

### Unit of analysis and handling of recurrent episodes

The primary analysis used pneumothorax episodes as the unit of analysis. This approach was chosen because treatment decisions and short-term outcomes were determined at the episode level. However, because recurrent episodes from the same patient may not be statistically independent and may influence the likelihood of surgery, we performed a predefined sensitivity analysis at the patient level. In this sensitivity analysis, only the first eligible spontaneous pneumothorax episode for each patient during the study period was retained. After this restriction, 256 independent patients remained, including 105 in the surgical group and 151 in the non-surgical group. Therefore, the 320-episode cohort represents the primary analytic cohort, whereas the 256-patient cohort represents the patient-level sensitivity cohort rather than a separate final inclusion number.

## Surgical and procedural approaches

### Chest tube drainage

All patients initially received chest drainage according to institutional practice. Drainage was maintained until clinical and radiological improvement was achieved, and tube removal was determined by the treating thoracic surgeon based on lung re-expansion, air-leak status, and drainage characteristics.

### Vats

VATS was performed under general anesthesia by the thoracic surgery team. The procedure generally consisted of thoracoscopic exploration, wedge resection of visible bullae or suspected leaking areas, and postoperative chest tube drainage. Because adjunctive pleurodesis or pleural abrasion was not consistently documented, these procedural details were not included in the main analysis.

### Data collection and analysis

Demographic and clinical data were extracted from electronic medical records. Demographic data included sex, age, height, weight, body mass index (BMI), and smoking history. Clinical data included pulmonary comorbidities (COPD, emphysema, and inactive pulmonary tuberculosis), treatment-related data (30-day follow-up chest CT, surgical approach, operative time, intraoperative blood loss, postoperative drainage time and volume, post-treatment complications, and hospital stay after the index procedure).

For the surgical group, postoperative blood tests obtained within 48 h after VATS were used. For the non-surgical group, blood tests obtained within 48 h after chest drainage or admission were used, according to the earliest available post-treatment laboratory record. SII, NLR, PLR, and LMR were calculated from complete blood count data.

Because the biological meaning of post-treatment blood tests may differ between treatment groups, biomarker timing was interpreted cautiously. In the surgical group, selected post-treatment blood tests primarily reflected the early postoperative inflammatory response after VATS, whereas in the non-surgical group, they reflected the early post-drainage or early post-admission inflammatory state. Therefore, between-group differences in inflammatory biomarkers may reflect not only treatment strategy, but also differences in sampling time, procedural trauma, perioperative medications, drainage course, and disease trajectory. Baseline pre-treatment inflammatory biomarkers were not consistently available for all episodes and therefore were not included in the primary adjusted analysis.

Pulmonary bullae were assessed on baseline chest CT and categorized as absent, isolated, or diffuse/multiloculated. This variable was recorded for baseline adjustment and was included as a covariate in the propensity-score model rather than analyzed as a primary exposure. Imaging assessment was performed independently by a thoracic surgeon (Qingguo Ding) and a thoracic radiologist (Peng Sun), with disagreements resolved by consensus.

Patients were categorized into three age groups: younger adults (15–44 years), middle-aged adults (45–64 years), and older adults (≥65 years).

Data collection and preliminary analyses were independently performed by two investigators (Qingguo Ding and Yingding Ruan), and subsequently verified by a third author (Jianwei Han) to ensure accuracy and reproducibility.

### Study outcomes

The primary outcome was defined as any thoracic complication within 30 days after discharge. We selected a discharge-based follow-up window because post-discharge outpatient visits, emergency revisits, readmissions, and follow-up chest CT examinations were consistently documented in the medical record system. This composite endpoint was designed to capture early thoracic events requiring continued observation, additional management, or clinical reassessment after the index treatment episode. Specifically, the composite included residual pneumothorax and/or pleural effusion requiring continued observation or treatment, persistent air leak, increased or worsening pneumothorax volume, recurrent pneumothorax, pulmonary infection, and intercostal neuralgia.

Given that these components differ in severity, timing, and clinical implications, we supplemented the composite outcome with a descriptive component-level breakdown. Clinically major thoracic events were defined as persistent air leak, increased or worsening pneumothorax, recurrent pneumothorax, and pulmonary infection, and were summarized separately. In contrast, residual pneumothorax and/or pleural effusion and intercostal neuralgia were interpreted more cautiously because they may represent minor postoperative or radiographic findings rather than events of equivalent severity to recurrence or persistent air leakage. Outcomes were identified from follow-up chest CT, outpatient visits, emergency revisits, or readmission records. Thirty-day all-cause mortality was also recorded. The secondary outcome was hospital stay after the index procedure.

### Statistical analysis

Stabilized inverse probability of treatment weighting (IPTW) based on propensity scores was applied to minimize confounding due to non-random treatment assignment and to control for group differences in baseline covariates. The propensity model included clinically relevant baseline covariates: sex, age, BMI, smoking history, pulmonary comorbidities, pneumothorax location, pulmonary bullae type, and pneumothorax volume. Stabilized weights were calculated by incorporating the marginal probability of treatment assignment into the numerator. To reduce the influence of extreme weights, truncation was applied at the 1st and 99th percentiles. The effective sample size (ESS) was calculated as (*Σ*w)^2/Σ(w^2), and the distribution of weights (including the maximum weight) was examined to assess weight stability. Covariate balance after IPTW was assessed using standardized mean differences (SMD), with SMD < 0.1 considered indicative of adequate balance.

Normally distributed continuous variables were analyzed using Student’s *t*-test and were presented as mean ± standard deviation. Non-normally distributed continuous variables were evaluated using the Wilcoxon rank-sum test and were expressed as median and interquartile range (25th–75th percentile). Categorical variables were compared using the Chi-square test or Fisher’s exact test and were presented as counts and percentages.

Treatment associations with 30-day post-discharge thoracic complications were assessed using IPTW-weighted logistic regression, and hospital stay after the index procedure was analyzed using IPTW-weighted linear regression. Robust sandwich standard errors were applied in the weighted models to account for variance estimation after weighting. Because the primary analysis was episode-based, we additionally calculated cluster-robust standard errors by patient identifier as a sensitivity analysis to account for within-patient correlation. Crude unweighted regression analyses were performed for descriptive comparison.

All statistical tests were two-sided, and a *p*-value of less than 0.05 was considered statistically significant. Statistical analysis was performed using R software (version 4.1.3; R Foundation for Statistical Computing, Vienna, Austria).

## Results

### Patient cohort and baseline characteristics

A total of 378 pneumothorax episodes were screened. After exclusion of non-spontaneous pneumothorax and other ineligible episodes, 320 spontaneous pneumothorax episodes were included in the primary episode-based analysis, including 120 episodes treated with VATS and 200 treated without surgery. Because some patients contributed more than one eligible episode, a patient-level sensitivity cohort was constructed by retaining only the first eligible spontaneous pneumothorax episode for each patient during the study period; this cohort included 256 independent patients, comprising 105 surgical and 151 non-surgical patients. The cohort comprised 293 males (91.6%) and 27 females (8.4%), and their age distribution was as follows: ≤44 years (38.1%), 45–64 years (21.9%), and ≥65 years (40.0%). Pulmonary comorbidities were present in 80 cases.

**Figure 1:**
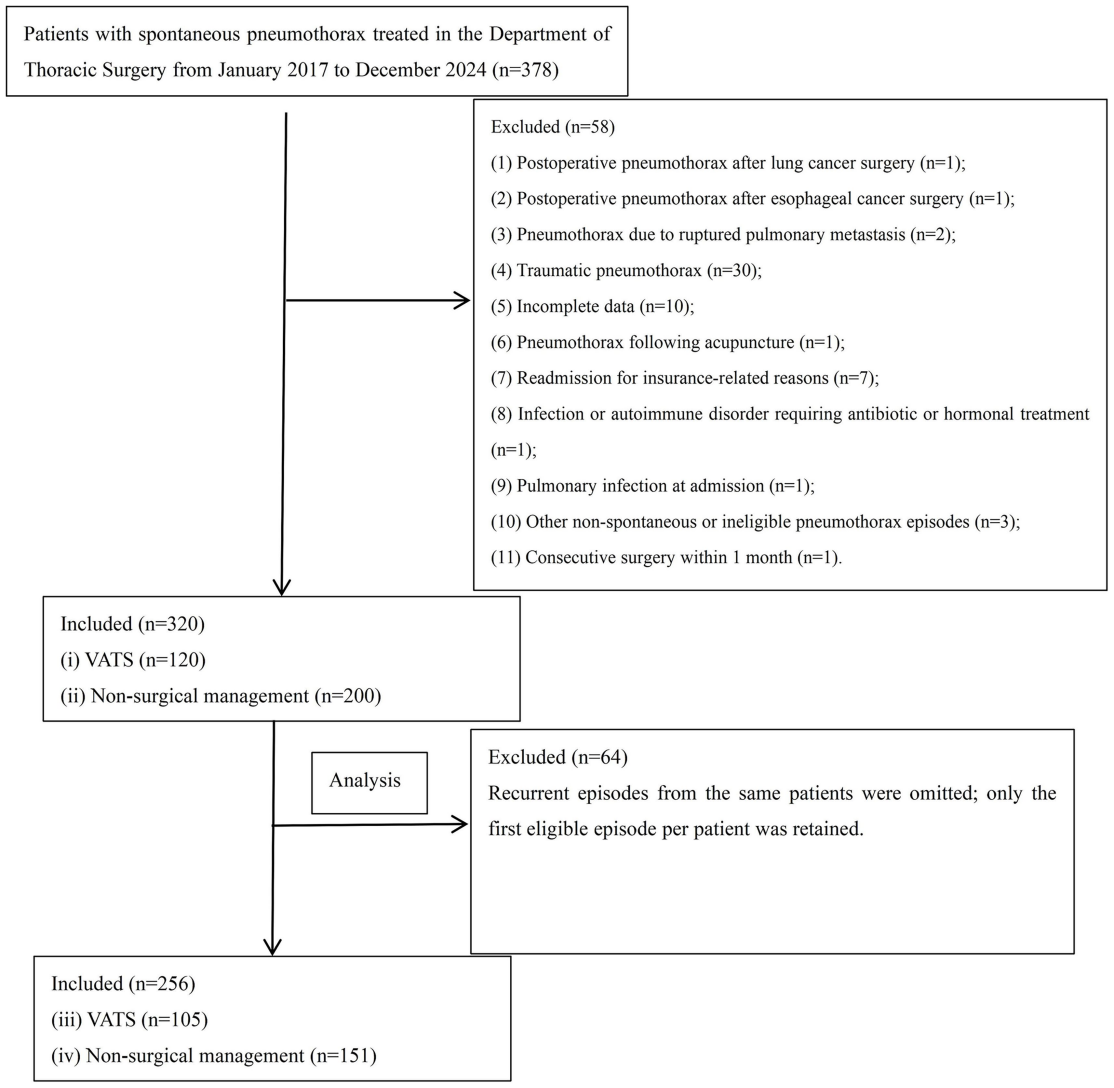
Flowchart of patient selection. A total of 378 pneumothorax episodes were treated in the Thoracic Department between January 2017 and December 2024. After applying exclusion criteria, 320 spontaneous pneumothorax episodes were included, comprising 120 managed with VATS and 200 managed with non-surgical management. For the patient-level sensitivity analysis, only the first eligible spontaneous pneumothorax episode for each patient during the study period was retained, resulting in 256 independent patients, including 105 managed with VATS and 151 managed with non-surgical management.

The flowchart of patient selection is shown in Figure 1. Baseline characteristics and covariate balance before and after IPTW in the episode-based cohort are presented in Table 1. After IPTW, balance was achieved across all measured baseline covariates, including sex, age distribution, BMI, pulmonary comorbidities, smoking history, pneumothorax location, pulmonary bullae type, and pneumothorax volume, with all SMDs < 0.1.

**Table 1:** Baseline characteristics and covariate balance before and after IPTW in the episode-based cohort.

Variables	Before IPTW			SMD	After IPTW			SMD
	Non-surgical management (*n* = 200)	VATS (*n* = 120)	*p*-value		Non-surgical management (Weighted)	VATS (Weighted)	*p*-value	
Sex, *n* (%)			0.640	0.055			0.844	−0.022
Male	182 (91.0)	111 (92.5)			184 (91.5)	106 (92.1)		
Female	18 (9.0)	9 (7.5)			17 (8.5)	9 (7.9)		
Smoking, *n* (%)	107 (53.5)	58 (48.3)	0.371	0.103	106 (52.6)	62 (53.9)	0.805	−0.026
Age, *n* (%)			<0.001	0.659*			0.668	−0.012
≤44 years	58 (29.0)	64 (53.3)			79 (39.0)	46 (39.6)		
≥45, ≤64	40 (20.0)	30 (25.0)			43 (21.1)	29 (24.8)		
≥65	102 (51.0)	26 (21.7)			80 (39.9)	41 (35.6)		
BMI, mean ± SD	19.31 ± 2.78	19.51 ± 2.92	0.552	0.069	19.43 ± 2.75	19.51 ± 2.90	0.781	0.028
Pulmonary comorbidities, *n* (%)	53 (26.5)	27 (22.5)	0.424	0.093	51 (25.5)	29 (25.4)	0.976	0.002
Location, *n* (%)			0.060	0.218			0.711	−0.050
Left	115 (57.5)	56 (46.7)			108 (53.5)	59 (51.1)		
Right	85 (42.5)	64 (53.3)			94 (46.5)	56 (48.9)		
Pulmonary bullae on baseline CT, *n* (%)			0.401	0.225			0.453	0.038
No	90 (45.0)	55 (45.8)			96 (47.8)	53 (45.9)		
Isolated	33 (16.5)	29 (24.2)			31 (15.6)	24 (20.9)		
Diffuse	77 (38.5)	36 (30.0)			74 (36.6)	38 (33.2)		
Estimated pneumothorax volume, median (P25, P75)	50.00 (40.00, 70.00)	60.00 (40.00, 75.00)	0.393	0.101	60.00 (40.00, 70.00)	60.00 (40.00, 70.00)	0.832	0.000

BMI, Body Mass Index; IPTW, Inverse Probability of Treatment Weighting; M (P25, P75), Median (25th percentile,75th percentile); SD, Standard Deviation; SMD, Standard Mean Difference; VATS, Video-Assisted Thoracoscopic Surgery.

Data are presented as *n* (%) for categorical variables and mean ± SD or median (P25, P75) for continuous variables.

SMD indicates the difference in means or proportions between groups divided by the pooled standard deviation. An SMD < 0.1 (10%) indicates adequate balance between groups.

Values in the “After IPTW” columns are weighted estimates. The effective sample size may differ from the original count due to weighting.

*For Age (categorical), the SMD represents the global difference across all categories.

The sum of the stabilized weights was 316.9, which was close to the original sample size (*N* = 320). The effective sample size (ESS) after weighting was 283 (88.4% of the original cohort), and the maximum individual weight was 2.24, indicating adequate weight stability.

After IPTW, the surgical group exhibited significantly higher systemic inflammatory indices than the non-surgical group, including SII (1583.28 [1088.57–2120.00] vs. 678.82 [432.90–1409.08], *p* < 0.001), PLR (205.43 [163.28–270.45] vs. 139.81 [105.67–210.00], *p* < 0.001), NLR (8.28 [6.17–11.50] vs. 3.61 [2.20–7.78], *p* < 0.001), and WBC (9.20 [7.80–11.70] vs. 7.08 [5.50–8.80], *p* < 0.001). Conversely, the surgical group presented significantly lower LMR (1.50 [1.00–2.50] vs. 3.00 [2.00–4.50], *p* < 0.001), albumin (34.91 ± 5.46 g/L vs. 41.31 ± 5.15 g/L, *p* < 0.001), and hemoglobin (124.01 ± 17.41 g/L vs. 132.56 ± 17.60 g/L, *p* < 0.001) (Table 2).

**Table 2:** Post-treatment inflammatory biomarkers and short-term outcomes before and after IPTW.

Variables	Before IPTW			SMD	After IPTW			SMD
	Non-surgical management (*n* = 200)	VATS (*n* = 120)	*p*-value		Non-surgical management (Weighted)	VATS (Weighted)	*p*-value	
Post-treatment SII, median (P25, P75)	747.25 (441.06, 1565.30)	1565.27 (1069.88, 2056.54)	<0.001	0.364	678.82 (432.93, 1421.11)	1583.28 (1090.07, 2119.09)	<0.001	0.382
Post-treatment PLR, median (P25, P75)	148.17 (108.34, 219.00)	195.05 (155.75, 253.91)	<0.001	0.087	139.81 (105.66, 209.86)	205.43 (163.12, 270.45)	<0.001	0.095
Post-treatment NLR, median (P25, P75)	4.11 (2.46, 9.39)	8.29 (6.18, 11.50)	<0.001	0.416	3.61 (2.20, 7.79)	8.28 (6.16, 11.50)	<0.001	0.425
Post-treatment LMR, median (P25, P75)	2.86 (1.67, 4.21)	1.50 (1.12, 2.11)	<0.001	1.061	3.00 (1.75, 4.67)	1.50 (1.10, 2.11)	<0.001	1.080
Post-treatment WBC, median (P25, P75)	7.10 (5.57, 8.80)	9.40 (7.97, 11.80)	<0.001	0.918	7.08 (5.50, 8.80)	9.20 (7.80, 11.70)	<0.001	0.925
Post-treatment ALB, mean ± SD	40.58 ± 5.16	35.86 ± 5.65	<0.001	0.876	41.31 ± 5.15	34.91 ± 5.46	<0.001	0.890
Post-treatment HGB, mean ± SD	130.98 ± 18.02	125.62 ± 16.64	0.008	0.310	132.56 ± 17.60	124.01 ± 17.41	<0.001	0.325
Drainage time, median (P25, P75)	3.00 (1.00, 5.00)	3.00 (2.00, 5.00)	0.597	0.034	3.00 (1.00, 5.00)	3.00 (2.00, 6.00)	0.005	0.045
Hospital stay after the index procedure, median (P25, P75)	4.00 (2.00, 6.00)	5.00 (3.00, 8.00)	0.001	0.146	3.00 (2.00, 6.00)	5.00 (3.00, 10.00)	<0.001	0.160
Antibiotics, *n* (%)			<0.001	0.434			<0.001	0.450
No	183 (91.5)	91 (75.8)			186 (92.3)	85 (73.8)		
Yes	17 (8.5)	29 (24.2)			15 (7.7)	30 (26.2)		
30-day post-discharge thoracic complications, *n* (%)			<0.001	0.435			<0.001	0.442
No	138 (69.0)	104 (86.7)			144 (71.5)	100 (86.8)		
Yes	62 (31.0)	16 (13.3)			57 (28.5)	15 (13.2)		

ALB, Albumin; HGB, Hemoglobin; IPTW, Inverse Probability of Treatment Weighting; SII, Systemic Immune Inflammation Index; LMR, Lymphocyte-to-Monocyte Ratio; M (P25, P75), Median (25th percentile,75th percentile); NLR, Neutrophil-to-Lymphocyte Ratio; PLR, Platelet-to-Lymphocyte Ratio; SD, Standard Deviation; SMD, Standard Mean Difference; VATS, Video-Assisted Thoracoscopic Surgery; WBC, White Blood Cell.

Data are presented as *n* (%) for categorical variables, mean ± SD for normally distributed continuous variables, and median (P25, P75) for skewed continuous variables.

Before IPTW: Unadjusted comparisons between Non-surgical management and VATS groups.

After IPTW: Weighted estimates derived from the inverse probability of treatment weighting model. These values represent outcomes in a pseudo-population where baseline confounders are balanced.

SMD Interpretation: In this table (outcomes), the SMD reflects the magnitude of the difference between groups after adjusting for confounders. Unlike baseline characteristics in Table 1, the SMDs in this table reflect the magnitude of between-group differences after weighting and should not be interpreted as causal effect estimates.

*p*-values were calculated using weighted *t*-tests, Wilcoxon rank-sum tests, or Chi-square tests as appropriate.

### Clinical outcomes

In descriptive analyses (Table 2), the surgical group demonstrated longer drainage duration (3.00 [2.00–6.00] days vs. 3.00 [1.00–5.00] days, *p* = 0.005), longer hospital stay after the index procedure (5.00 [3.00–10.00] days vs. 3.00 [2.00–6.00] days, *p* < 0.001), higher rates of antibiotic use (26.2% vs. 7.7%, *p* < 0.001), and lower rates of any 30-day post-discharge thoracic complication (13.2% vs. 28.5%, *p* < 0.001). The overall rate and detailed composition of 30-day post-discharge thoracic complications are shown in Table 3.

**Table 3:** Components of 30-day post-discharge thoracic complications.

Complication type	Non-surgical management (*n* = 62)	VATS (*n* = 16)	Total (*n* = 78)
Residual pneumothorax and/or pleural effusion	14 (22.6%)	15 (93.8%)	29 (37.2%)
Persistent air leak	25 (40.3%)	0 (0.0%)	25 (32.1%)
Increased pneumothorax volume/worsening pneumothorax	5 (8.1%)	0 (0.0%)	5 (6.4%)
Recurrent pneumothorax	9 (14.5%)	0 (0.0%)	9 (11.5%)
Pulmonary infection	9 (14.5%)	0 (0.0%)	9 (11.5%)
Intercostal neuralgia	0 (0.0%)	1 (6.2%)	1 (1.3%)

Percentages were calculated among episodes with any 30-day post-discharge thoracic complication within each treatment group. Because the components differed in clinical severity, this table was used as a descriptive component-level breakdown of the composite endpoint.

Recurrent episodes were not excluded from the primary episode-based analysis. To assess whether repeated episodes influenced the results, we performed a sensitivity analysis restricted to the first eligible spontaneous pneumothorax episode per patient. After this restriction, 256 independent patients remained; the overall characteristics and covariate balance of this patient-level sensitivity cohort are presented in Supplementary Tables S1, S2.

In the total cohort, the surgical group had 16 complications (15 cases of residual pneumothorax or pleural effusion and one intercostal neuralgia), while the non-surgical group had 62 complications (11 cases of residual pneumothorax, 25 persistent air leaks, five cases of increased pneumothorax volume, nine cases of recurrent pneumothorax, nine pulmonary infections, and three pleural effusions) within 30 days after discharge (Table 3).

In the patient-level sensitivity cohort, in which only the first eligible spontaneous pneumothorax episode per patient was retained, 13 surgical patients and 44 non-surgical patients experienced 30-day post-discharge thoracic complications. The reduction in the number of complications compared with the primary cohort reflects the removal of repeated episodes from patients with recurrent pneumothorax, not *post hoc* exclusion of complications.

### Primary outcomes

After IPTW adjustment, IPTW-weighted logistic regression showed that VATS was associated with a significantly lower risk of 30-day post-discharge thoracic complications (OR = 0.38, 95% CI = 0.21–0.71, *p* = 0.002; Table 4). This finding was consistent in the patient-level sensitivity cohort (Supplementary Table S3) and remained significant when cluster-robust standard errors by patient identifier were used (OR = 0.38, 95% CI = 0.20–0.73, *p* = 0.004; Supplementary Table S4).

**Table 4:** Crude and IPTW-weighted associations with 30-day post-discharge thoracic complications.

Variables	Crude OR (95% CI)	*p*-value	IPTW-weighted OR (95% CI)	*p*-value
Group				
Non-surgical management	Ref		Ref	
VATS	0.34 (0.19, 0.63)	<0.001	0.38 (0.21, 0.71)	0.002
Sex				
Male	Ref			
Female	0.88 (0.34, 2.26)	0.786		
Smoking				
No	Ref			
Yes	1.29 (0.77, 2.16)	0.325		
Age				
≤44	Ref			
≥45, ≤64	0.26 (0.13, 0.50)	<0.001		
≥65	1.18 (0.63, 2.21)	0.605		
BMI	0.95 (0.86, 1.04)	0.275		
Pulmonary comorbidities				
No	Ref			
Yes	1.89 (1.08, 3.30)	0.025		
Location				
Left	Ref			
Right	1.05 (0.63, 1.75)	0.859		
Pulmonary bullae				
No	Ref			
Isolated	0.49 (0.28, 0.87)	0.015		
Diffuse	1.84 (0.89, 3.80)	0.102		
Pneumothorax volume	1.01 (0.99, 1.02)	0.107		
Post-treatment SII	1.00 (0.99, 1.01)	0.511		
Post-treatment PLR	1.00 (0.99, 1.01)	0.197		
Post-treatment NLR	1.01 (0.97, 1.05)	0.613		
Post-treatment LMR	1.09 (0.94, 1.27)	0.252		
Post-treatment WBC	0.96 (0.88, 1.05)	0.413		
Post-treatment ALB	0.99 (0.95, 1.04)	0.837		
Post-treatment HGB	0.99 (0.98, 1.01)	0.278		
Drainage time	1.10 (1.04, 1.15)	<0.001		
Hospital stay after the index procedure	1.08 (1.04, 1.13)	<0.001		
Antibiotics				
No	Ref			
Yes	1.44 (0.72, 2.86)	0.303		

OR, Odds Ratio; CI, Confidence interval; IPTW, Inverse Probability of Treatment Weighting; SII, Systemic Immune-inflammation Index; PLR, Platelet-to-Lymphocyte Ratio; NLR, Neutrophil-to-Lymphocyte Ratio; LMR, Lymphocyte-to-Monocyte Ratio; VATS, Video-Assisted Thoracoscopic Surgery; WBC, White Blood Cell count; ALB, Albumin; HGB, Hemoglobin.

The crude column presents odds ratios derived from standard logistic regression without weighting. These crude estimates are descriptive and should not be interpreted as mutually adjusted effects. The IPTW-weighted model estimated the average treatment effect of VATS after balancing measured baseline covariates. Baseline covariates were not re-entered into the weighted outcome model to avoid overadjustment. Robust sandwich standard errors were used for the IPTW-weighted model. Cluster-robust standard errors by patient identifier were additionally examined as a sensitivity analysis and are presented in Supplementary Table S4. For continuous variables, odds ratios represent the change in odds per 1-unit increase in the original measurement scale. Reference groups were non-surgical management, male sex, no smoking, age ≤44 years, no pulmonary comorbidities, left-sided pneumothorax, no pulmonary bullae, and no antibiotic use.

### Secondary outcomes

In IPTW-weighted linear regression, VATS was associated with significantly longer hospital stay after the index procedure (*β* = 1.91 days, 95% CI = 0.34–3.49, *p* = 0.017; Table 5). A similar association was observed in the patient-level sensitivity cohort (Supplementary Table S5) and remained significant when cluster-robust standard errors by patient identifier were used (*β* = 1.91 days, 95% CI = 0.33–3.50, *p* = 0.018; Supplementary Table S4).

**Table 5:** Crude and IPTW-weighted associations with hospital stay after the index procedure.

Variables	Crude *β* (95% CI)	*p*-value	IPTW-weighted *β* (95% CI)	*p*-value
Group				
Non-surgical management	Ref		Ref	
VATS	0.99 (−0.57, 2.56)	0.213	1.91 (0.34, 3.49)	0.017
Sex				
Male	Ref			
Female	−1.97 (−4.69, 0.76)	0.156		
Smoking				
No	Ref			
Yes	3.11 (1.63, 4.59)	<0.001		
Age				
≤44	Ref			
≥45, ≤64	2.60 (0.66, 4.55)	0.009		
≥65	4.62 (2.98, 6.27)	<0.001		
BMI	−0.04 (−0.31, 0.23)	0.757		
Pulmonary comorbidities				
No	Ref			
Yes	2.82 (1.10, 4.55)	0.001		
Location				
Left	Ref			
Right	0.53 (−0.99, 2.05)	0.492		
Pulmonary bullae				
No	Ref			
Isolated	−0.01 (−2.04, 2.03)	0.99		
Diffuse	2.40 (0.72, 4.09)	0.005		
Pneumothorax volume	0.04 (0.00, 0.07)*	0.049		
Post-treatment SII	0.01 (0.01, 0.02)**	<0.001		
Post-treatment PLR	0.01 (0.01, 0.02)	<0.001		
Post-treatment NLR	0.17 (0.06, 0.28)	0.002		
Post-treatment LMR	−0.49 (−0.96, −0.03)	0.039		
Post-treatment WBC	0.13 (−0.14, 0.39)	0.352		
Post-treatment ALB	−0.33 (−0.46, −0.21)	<0.001		
Post-treatment HGB	−0.08 (−0.12, −0.03)	<0.001		
Drainage time	0.97 (0.93, 1.01)	<0.001		
Antibiotics				
No	Ref			
Yes	8.36 (6.40, 10.31)	<0.001		

*β*, unstandardized regression coefficient; CI, Confidence Interval; IPTW, Inverse Probability of Treatment Weighting.

Outcome variable was hospital stay after the index procedure, measured in days. The crude column presents unweighted associations between each listed variable and hospital stay using standard linear regression. These crude estimates are descriptive and should not be interpreted as mutually adjusted effects. The IPTW-weighted model estimated the average treatment effect of VATS after balancing measured baseline covariates. Baseline covariates were not re-entered into the weighted outcome model to avoid overadjustment. Robust sandwich standard errors were used for the IPTW-weighted model. Cluster-robust standard errors by patient identifier were additionally examined as a sensitivity analysis and are presented in Supplementary Table S4. Regression coefficients represent the change in hospital stay in days. For estimated pneumothorax volume, the coefficient represents the change in hospital stay per 1-unit increase in the recorded volume variable. For inflammatory indices, coefficients represent the change per 1-unit increase. * Coefficient represents change in hospital stay per 1 mL (or your unit) increase in volume. ** For inflammatory indices (SII, PLR, NLR), coefficients represent change per 1-unit increase.

In exploratory analyses stratified by pulmonary comorbidity, the direction of the treatment association was generally consistent with the overall analysis for both 30-day post-discharge thoracic complications and hospital stay after the index procedure. However, there was no statistically significant interaction between treatment group and pulmonary comorbidity for either outcome (P for interaction = 0.142 and 0.475, respectively; Supplementary Table S4). These subgroup results should therefore be interpreted as exploratory.

## Discussion

In this episode-based retrospective cohort of spontaneous pneumothorax, VATS was associated with fewer 30-day post-discharge thoracic complications but longer hospitalization compared with non-surgical management. This association persisted after stabilized IPTW adjustment, with VATS associated with an approximately 60% lower odds of the composite 30-day post-discharge thoracic complication endpoint. The estimates were similar when robust sandwich standard errors and patient-level clustered standard errors were used. The direction of effect was also preserved in the patient-level sensitivity cohort restricted to the first eligible spontaneous pneumothorax episode per patient. Because the primary outcome combined events of different clinical severity, these results should be interpreted as evidence of an association with fewer early composite thoracic events rather than a uniform reduction in all clinically important complications. Overall, the findings suggest a clinically relevant trade-off: VATS was associated with fewer early thoracic events, mainly those related to ongoing air leakage or early recurrence, but also with greater post-treatment inflammatory response and longer hospital stay.

The component-level distribution of the primary outcome is important for interpreting this association. After IPTW, the overall 30-day post-discharge complication rate was lower in the VATS group than in the non-surgical group (13.2% vs. 28.5%). However, this difference should not be interpreted as a uniform reduction across all complication types. In the primary cohort, persistent air leak, increased or worsening pneumothorax volume, recurrent pneumothorax, and pulmonary infection occurred predominantly in the non-surgical group, whereas complications in the VATS group were mainly residual pneumothorax and/or pleural effusion. Residual postoperative pleural findings are not clinically equivalent to recurrent pneumothorax or persistent air leakage. Therefore, the apparent benefit associated with VATS appears to be driven mainly by fewer ongoing air-leak-related events, early recurrence, worsening pneumothorax, and pulmonary infection, rather than by elimination of all minor radiographic abnormalities. Mechanistically, thoracoscopic exploration and wedge resection of visible bullae or suspected leaking areas may directly address the anatomical source of persistent air leakage (16, 17). Previous studies have also reported lower recurrence rates after VATS than after non-surgical strategies (2, 18–21), supporting the clinical plausibility of this component-specific pattern.

Several randomized trials provide useful context for these findings. In the PSP trial, conservative management was non-inferior to interventional management for lung re-expansion in selected patients with primary spontaneous pneumothorax and resulted in fewer serious adverse events (22). The RAMPP trial also showed that ambulatory management reduced total hospital stay compared with standard care, although careful patient selection and safety monitoring remained important (23). Together with recent guideline recommendations, these data support a less invasive approach for clinically stable patients rather than routine escalation to surgery (24). Our findings should therefore not be interpreted as evidence that VATS is preferable for all patients with spontaneous pneumothorax. Rather, they suggest that in real-world practice, where surgery is often chosen for patients with recurrence risk, persistent air leak, radiological bullae, or concern for early treatment failure, VATS may be associated with fewer short-term thoracic complications at the cost of longer hospitalization and greater treatment-related inflammatory burden.

Randomized surgical studies also indicate that the benefit of operative management may depend on the accompanying pleural procedure. In high-risk primary spontaneous pneumothorax, recurrence-prevention strategies have been evaluated using different thoracoscopic pleurodesis approaches (25). However, the benefit of adding pleural intervention is not uniform across procedures. In a randomized trial, mechanical pleurodesis did not significantly reduce recurrence compared with wedge resection alone, while intraoperative bleeding and postoperative pleural drainage were higher (26). A prospective randomized comparison of uniportal, two-port, and three-port VATS further suggested that different minimally invasive approaches may achieve broadly comparable clinical outcomes while differing in recovery profiles (27). This broader spectrum of pleural intervention is highly relevant to our study. The lower rate of early thoracic complications after VATS in our cohort may reflect the combined effects of wedge resection, possible pleural intervention, postoperative drainage management, and treatment selection. Because pleurodesis and pleural abrasion were not consistently documented, however, we could not isolate which specific surgical component contributed most to the observed association.

Beyond clinical outcomes, the higher post-treatment inflammatory biomarker levels observed after VATS should be interpreted with caution. In our cohort, VATS was associated with higher SII, PLR, NLR, and WBC levels, but lower LMR, albumin, and hemoglobin. For example, after IPTW, SII was markedly higher in the VATS group than in the non-surgical group, and similar directional differences were observed for NLR and PLR. This pattern likely reflects a systemic stress response after thoracic surgery, characterized by neutrophil predominance, relative lymphocyte suppression, platelet activation, and transient metabolic or nutritional stress. Importantly, this inflammatory response was not accompanied by an increased rate of pulmonary infection. Therefore, elevated SII, NLR, PLR, or WBC after VATS should not be interpreted simply as evidence of infection. In SP, these markers may reflect treatment-related inflammatory burden more than treatment failure or septic complications.

The apparent paradox—elevated inflammatory indices coupled with reduced thoracic complications—challenges the conventional reflex to equate high biomarkers with poor outcomes. This distinction is central to interpreting our results. In lung cancer surgery, perioperative inflammatory indices have been associated with postoperative pneumonia, atrial fibrillation, and short-term pulmonary imaging changes (14, 15). In contrast, in this SP cohort, elevated inflammatory markers coexisted with improved short-term thoracic outcomes. This distinction is important because inflammatory biomarkers in oncologic thoracic surgery are often interpreted in relation to postoperative infection, tumor biology, or long-term prognosis, whereas in SP the immediate clinical priority is control of air leakage, lung re-expansion, and prevention of early recurrence. Thus, biomarker interpretation in SP should be integrated with clinical status, imaging findings, air-leak persistence, drainage course, and recurrence risk, rather than used in isolation.

The reduction in LMR in the VATS group also deserves attention. Previous studies have suggested that monocyte- and lymphocyte-based indices may be associated with recurrence tendency or disease activity in SP (28, 29). Saricam et al. reported that LMR differed between recurrent and non-recurrent SP cases, supporting a possible link between systemic immune balance and recurrence risk (29). In addition, emerging evidence suggests that pneumothorax pathogenesis may involve pleural-level inflammatory mechanisms beyond visible bullae alone (30, 31). However, our findings should not be overextended. In the present study, LMR was evaluated as part of a post-treatment inflammatory profile, not as an independent long-term recurrence predictor. We did not establish biomarker cutoffs, construct a biomarker-based prediction model, or assess long-term recurrence. Therefore, the LMR finding should be considered hypothesis-generating.

The timing of surgical intervention remains clinically important. Although many first episodes of SP can be managed without surgery, VATS may be considered when recurrence prevention is prioritized or when patients have persistent air leak, recurrent pneumothorax, extensive bullous disease, or specific occupational requirements (22, 32). In our cohort, the lower complication rate in the VATS group must be weighed against longer hospitalization and greater inflammatory response. This is clinically relevant: for patients at high risk of persistent air leak or early recurrence, the short-term burden of surgery may be justified; for lower-risk patients, non-surgical management may remain appropriate. For younger patients in high-risk occupations, such as pilots or divers, or for individuals with limited access to emergency care, even a single recurrence may carry disproportionate consequences; therefore, the threshold for discussing surgical options may be lower, potentially even after an early episode.

Adjunctive pleural procedures remain an important source of heterogeneity in surgical management. Although previous studies suggest that VATS combined with selected pleural interventions may reduce recurrence risk (20, 33–38), pleurodesis and pleural abrasion were not consistently documented in our cohort. Therefore, the lower rate of early thoracic complications observed after VATS should be interpreted as the effect of a real-world surgical strategy rather than wedge resection alone. Future studies should record pleurodesis status, pleural abrasion, staple-line coverage, drainage protocols, and extent of resection more systematically.

Age, pulmonary comorbidities, and bullous changes also appear clinically relevant. Older patients and those with COPD, emphysema, or previous pulmonary tuberculosis may have reduced pulmonary reserve, delayed lung re-expansion, and a higher likelihood of prolonged drainage (39–42). In our crude analyses, pulmonary comorbidity was associated with longer hospital stay, supporting the clinical impression that secondary or comorbidity-associated SP requires more cautious monitoring. Similarly, diffuse bullae may indicate broader structural lung vulnerability and a higher probability of persistent or recurrent air leakage. However, in this study, bullae classification was used mainly as a baseline covariate in the propensity-score model rather than as a primary exposure. Therefore, the relationship between bullae pattern and clinical outcomes should be interpreted cautiously.

This study has several strengths. First, the use of stabilized IPTW improved comparability between the surgical and non-surgical groups and reduced measured baseline imbalance. Second, weight diagnostics, including the effective sample size and maximum individual weight, supported the stability of the weighted analysis. In addition, robust sandwich standard errors and cluster-robust standard errors by patient identifier were examined, and the primary findings remained stable. Third, the patient-level sensitivity analysis restricted to the first eligible spontaneous pneumothorax episode for each patient during the study period addressed the concern that recurrent episodes from the same patient might influence the primary episode-based results. Finally, the study combined treatment strategy, inflammatory biomarkers, and clinically meaningful 30-day thoracic outcomes, providing a pragmatic picture of early recovery after SP management.

Several limitations should also be acknowledged. First, the retrospective design remains vulnerable to selection bias, residual confounding, and incomplete documentation. Second, the primary analysis was episode-based. Although this approach is clinically relevant because treatment decisions and short-term outcomes occur at the episode level, repeated episodes from the same patient may introduce within-patient correlation. We therefore performed a patient-level sensitivity analysis, but this approach cannot fully replace prospective patient-level follow-up. Third, treatment allocation was not random. Patients selected for VATS may have had recurrent disease, persistent air leak, more visible bullae, or stronger preference for recurrence prevention, which may not be fully captured in the available covariates. Fourth, the cohort included both primary and secondary or comorbidity-associated spontaneous pneumothorax, and a clear distinction between PSP and SSP could not be made for all retrospective episodes. Although pulmonary comorbidity was adjusted for as a baseline covariate, this variable may not fully account for the clinical heterogeneity between PSP and SSP. Therefore, subgroup differences by pneumothorax subtype should be evaluated in future prospective studies. Fifth, adjunctive procedural details, including pleurodesis, pleural abrasion, and stapling extent, were not consistently documented, limiting our ability to determine which specific surgical components contributed most to the observed outcome differences. Finally, follow-up was limited to 30 days after discharge, and long-term recurrence could not be evaluated.

Future studies should focus on identifying which patients are most likely to benefit from early VATS and which can be safely managed with conservative, ambulatory, or drainage-based strategies. Prospective multicenter studies with patient-level follow-up are needed, with stratification by primary versus secondary spontaneous pneumothorax, first episode versus recurrence, persistent air leak, bullae pattern, pulmonary comorbidity, and patient preference. Surgical details should be documented more consistently, including wedge resection, pleurodesis or pleural abrasion, staple-line coverage, drainage protocols, analgesic use, and perioperative antibiotics. Serial measurement of inflammatory biomarkers before treatment, after intervention, at discharge, and during follow-up may also help distinguish transient surgical stress from infection, persistent air leak, recurrence, or delayed recovery. Combining clinical features, CT findings, treatment details, air-leak course, and biomarker changes may eventually support more individualized treatment decisions in spontaneous pneumothorax.

In conclusion, in this retrospective episode-based cohort of SP, VATS was associated with fewer 30-day post-discharge thoracic complications but longer hospitalization compared with non-surgical management. Post-treatment inflammatory biomarkers were higher in the VATS group, but this pattern likely reflected surgical stress rather than infection alone. These findings support a balanced interpretation of VATS in SP: short-term thoracic outcomes may improve, but at the cost of greater treatment-related inflammatory burden and longer hospital stay.

## Data Availability

The datasets presented in this article are not readily available because any researchers interested in this study could contact QD to request the data. Requests to access the datasets should be directed to dingqingguo@sina.com.

## Glossary

ALB: albumin
BMI: body mass index
CI: confidence interval
COPD: chronic obstructive pulmonary disease
CT: computed tomography
ESS: effective sample size
HGB: hemoglobin
IPTW: inverse probability of treatment weighting
IQR: interquartile range
LMR: lymphocyte-to-monocyte ratio
M (P25: P75) median (25th percentile–75th percentile)
NLR: neutrophil-to-lymphocyte ratio
OR: odds ratio
PLR: platelet-to-lymphocyte ratio
PSP: primary spontaneous pneumothorax
SD: standard deviation
SII: systemic immune-inflammation index
SMD: standardized mean difference
SP: spontaneous pneumothorax
VATS: video-assisted thoracoscopic surgery
WBC: white blood cell count
