# Supplementary material for: Inflammatory biomarkers and 30-day thoracic outcomes after surgical versus non-surgical management of spontaneous pneumothorax: a retrospective cohort study
Source: Front Med (Lausanne). 2026 Jul 3;13:1868899. doi: 10.3389/fmed.2026.1868899 (PMC13375800; doi:10.3389/fmed.2026.1868899)
Supplement: Supplementary file 1 [file Table_1.DOCX]

| Supplementary Table S1. Overall characteristics of the patient-level sensitivity cohort before and after IPTW weighting | | |
| --- | --- | --- |
| Variables | Before IPTW | After IPTW |
| Group, n(%) |  |  |
| Non-surgical management | 151 (59.0) | 152 (59.8) |
| VATS | 105 (41.0) | 102 (40.2) |
| Sex, n(%) |  |  |
| Male | 231 (90.2) | 230 (90.6) |
| Female | 25 (9.8) | 24 (9.4) |
| Smoking, n(%) | 129 (50.4) | 132 (52.0) |
| Age, n(%) |  |  |
| ≤44 | 104 (40.6) | 105 (41.5) |
| ≥45, ≤ 64 | 58 (22.7) | 60 (23.5) |
| ≥65 | 94 (36.7) | 89 (35.0) |
| BMI, mean ± SD | 19.45 ± 2.94 | 19.53 ± 2.91 |
| Pulmonary Comorbidities, n(%) | 54 (21.1) | 52 (20.4) |
| Location, n(%) |  |  |
| Left | 128 (50.0) | 124 (49.0) |
| Right | 128 (50.0) | 129 (51.0) |
| Pulmonary Bullae, n(%) |  |  |
| No | 129 (50.4) | 132 (51.9) |
| Isolated | 47 (18.4) | 43 (17.1) |
| Diffuse | 80 (31.3) | 79 (31.0) |
| Pneumothorax Volume, median (P25, P75) | 60.00 (40.00, 73.75) | 60.00 (40.00, 70.00) |
| Post-treatment SII, median (P25, P75) | 1030.29 (556.48, 1802.98) | 1004.80 (536.50, 1745.72) |
| Post-treatment PLR, median (P25, P75) | 171.49 (120.23, 239.75) | 168.95 (119.89, 235.69) |
| Post-treatment NLR, median (P25, P75) | 6.15 (3.26, 9.96) | 5.96 (3.19, 9.65) |
| Post-treatment LMR, median (P25, P75) | 2.08 (1.33, 3.33) | 2.20 (1.35, 3.75) |
| Post-treatment WBC, median (P25, P75) | 7.80 (6.30, 10.06) | 7.80 (6.30, 10.00) |
| Post-treatment ALB, mean ± SD | 38.65 ± 5.99 | 38.87 ± 6.17 |
| Post-treatment HGB, mean ± SD | 128.52 ± 18.18 | 128.99 ± 18.40 |
| Drainage time, median (P25, P75) | 3.00 (2.00, 5.00) | 3.00 (2.00, 5.00) |
| Hospital stay after the index procedure, median (P25, P75) | 4.00 (3.00, 7.00) | 4.00 (3.00, 7.00) |
| Antibiotics, n (%) |  |  |
| No | 222 (86.7) | 221 (87.3) |
| Yes | 34 (13.3) | 32 (12.7) |
| 30-day post-discharge thoracic complications, n (%) |  |  |
| No | 199 (77.7) | 199 (78.7) |
| Yes | 57 (22.3) | 54 (21.3) |
| ALB, Albumin; BMI, Body Mass Index; HGB, Hemoglobin; IPTW, Inverse Probability of Treatment Weighting; SII, Systemic Immune Inflammation Index; LMR, Lymphocyte-to-Monocyte Ratio; M (P25,P75), Median (25th percentile,75th percentile); NLR, Neutrophil-to-Lymphocyte Ratio; PLR, Platelet-to-Lymphocyte Ratio; SD, Standard Deviation; VATS, Video-Assisted Thoracoscopic Surgery; WBC, White Blood Cell. Note: The patient-level sensitivity cohort included 256 independent patients after retaining only the first eligible spontaneous pneumothorax episode for each patient during the study period. Values in the “After IPTW weighting” column represent weighted estimates. | | |
