# Supplementary material for: Inflammatory biomarkers and 30-day thoracic outcomes after surgical versus non-surgical management of spontaneous pneumothorax: a retrospective cohort study
Source: Front Med (Lausanne). 2026 Jul 3;13:1868899. doi: 10.3389/fmed.2026.1868899 (PMC13375800; doi:10.3389/fmed.2026.1868899)
Supplement: Supplementary file 2 [file Table_2.DOCX]

| Supplementary Table S2. Baseline characteristics, post-treatment biomarkers, and outcomes in the patient-level sensitivity cohort before and after IPTW | | | | | | | | |
| --- | --- | --- | --- | --- | --- | --- | --- | --- |
| Variables | Before IPTW | | | SMD | After IPTW | | | SMD |
|  | Non-surgical management (n=151) | VATS (n=105) | P-Value |  | Non-surgical management  (Weighted) | VATS  (Weighted) | P-Value |  |
| Sex, n(%) |  |  | 0.164 | 0.182 |  |  | 0.780 | −0.031 |
| Male | 133 (88.1) | 98 (93.3) |  |  | 137 (90.2) | 93 (91.2) |  |  |
| Female | 18 (11.9) | 7 (6.7) |  |  | 15 (9.8) | 9 (8.8) |  |  |
| Smoking, n(%) | 76 (50.3) | 53 (50.5) | 0.982 | 0.003 | 78 (51.5) | 54 (52.7) | 0.799 | −0.024 |
| Age, n(%) |  |  | 0.001 | 0.510 |  |  | 0.305 | 0.041 |
| ≤44 years | 52 (34.4) | 52 (49.5) |  |  | 64 (42.3) | 41 (40.4) |  |  |
| ≥45, ≤ 64 | 29 (19.2) | 29 (27.6) |  |  | 31 (20.4) | 29 (28.0) |  |  |
| ≥65 | 70 (46.4) | 24 (22.9) |  |  | 57 (37.3) | 32 (31.6) |  |  |
| BMI, mean ± SD | 19.31 ± 2.85 | 19.66 ± 3.07 | 0.345 | 0.120 | 19.51 ± 2.87 | 19.56 ± 2.97 | 0.878 | 0.017 |
| Pulmonary Comorbidities, n(%) | 30 (19.9) | 24 (22.9) | 0.564 | 0.073 | 31 (20.5) | 21 (20.3) | 0.991 | 0.002 |
| Location, n(%) |  |  | 0.057 | 0.244 |  |  | 0.788 | 0.040 |
| Left | 83 (55.0) | 45 (42.9) |  |  | 75 (49.8) | 49 (47.9) |  |  |
| Right | 68 (45.0) | 60 (57.1) |  |  | 76 (50.2) | 53 (52.1) |  |  |
| Pulmonary Bullae, n(%) |  |  | 0.379 | 0.285 |  |  | 0.276 | 0.074 |
| No | 82 (54.3) | 47 (44.8) |  |  | 81 (53.5) | 51 (49.6) |  |  |
| Isolated | 21 (13.9) | 26 (24.8) |  |  | 21 (13.9) | 22 (21.7) |  |  |
| Diffuse | 48 (31.8) | 32 (30.4) |  |  | 49 (32.6) | 29 (28.6) |  |  |
| Pneumothorax Volume, median (P25, P75) | 50.00 (40.00, 70.00) | 60.00 (40.00, 75.00) | 0.350 | 0.121 | 60.00 (40.00, 71.28) | 60.00 (40.00, 70.00) | 0.580 | 0.000 |
| Post-treatment SII, median (P25, P75) | 683.73 (435.10, 1321.67) | 1580.13 (1069.50, 2019.13) | <0.001 | 0.451 | 671.58 (433.06, 1193.82) | 1583.28 (1066.08, 2065.50) | <0.001 | 0.463 |
| Post-treatment PLR, median (P25, P75) | 142.00 (106.23, 210.00) | 198.75 (160.14, 253.73) | <0.001 | 0.154 | 136.76 (104.60, 193.85) | 199.81 (163.43, 254.44) | <0.001 | 0.162 |
| Post-treatment NLR, median (P25, P75) | 3.61 (2.26, 7.67) | 8.57 (6.29, 11.50) | <0.001 | 0.558 | 3.57 (2.20, 7.12) | 8.32 (6.17, 11.50) | <0.001 | 0.569 |
| Post-treatment LMR, median (P25, P75) | 3.00 (1.75, 4.58) | 1.50 (1.12, 2.00) | <0.001 | 1.154 | 3.00 (1.85, 4.67) | 1.50 (1.11, 2.05) | <0.001 | 1.168 |
| Post-treatment WBC, median (P25, P75) | 6.90 (5.50, 8.70) | 9.30 (7.86, 11.70) | <0.001 | 0.859 | 7.08 (5.50, 8.80) | 9.10 (7.80, 11.70) | <0.001 | 0.845 |
| Post-treatment ALB, mean ± SD | 40.77 ± 5.35 | 35.59 ± 5.55 | <0.001 | 0.954 | 41.47 ± 5.21 | 35.00 ± 5.43 | <0.001 | 0.968 |
| Post-treatment HGB, mean ± SD | 130.83 ± 18.47 | 125.20 ± 17.29 | 0.015 | 0.316 | 132.31 ± 17.83 | 124.05 ± 18.21 | <0.001 | 0.328 |
| Drainage time, median (P25, P75) | 3.00 (1.00, 5.00) | 3.00 (2.00, 5.00) | 0.240 | 0.022 | 3.00 (1.00, 4.00) | 3.00 (2.00, 5.00) | 0.155 | 0.028 |
| Hospital stay after the index procedure, median (P25, P75) | 3.00 (2.00, 5.50) | 5.00 (3.00, 8.00) | <0.001 | 0.187 | 3.00 (2.00, 5.00) | 5.00 (3.00, 9.00) | <0.001 | 0.195 |
| Antibiotics, n (%) |  |  | 0.001 | 0.423 |  |  | <0.001 | 0.435 |
| No | 140 (92.7) | 82 (78.1) |  |  | 142 (93.8) | 79 (77.5) |  |  |
| Yes | 11 (7.3) | 23 (21.9) |  |  | 9 (6.2) | 23 (22.5) |  |  |
| 30-day post-discharge thoracic complications, n (%) |  |  | 0.002 | 0.422 |  |  | 0.002 | 0.430 |
| No | 107 (70.9) | 92 (87.6) |  |  | 110 (72.3) | 90 (88.0) |  |  |
| Yes | 44 (29.1) | 13 (12.4) |  |  | 42 (27.7) | 12 (12.0) |  |  |
| BMI, Body Mass Index; IPTW, Inverse Probability of Treatment Weighting; LMR, Lymphocyte-to-Monocyte Ratio; NLR, Neutrophil-to-Lymphocyte Ratio; PLR, Platelet-to-Lymphocyte Ratio; SII, Systemic Immune-Inflammation Index; SMD, Standardized Mean Difference; VATS, Video-Assisted Thoracoscopic Surgery; WBC, White Blood Cell. Notes: Data are presented as n (%) for categorical variables, mean ± SD for normally distributed continuous variables, and median (P25, P75) for skewed continuous variables. Before IPTW: Unadjusted comparisons between Non-surgical management and VATS groups. After IPTW: Weighted estimates derived from the inverse probability of treatment weighting model. Values represent outcomes in a pseudo-population where measured baseline covariates are balanced (all baseline SMD < 0.1). SMD Interpretation: For baseline characteristics, SMD < 0.1 indicates adequate balance. For post-treatment biomarkers and outcomes, the SMD reflects the magnitude of between-group differences after weighting and should not be interpreted as a causal effect estimate. P values were calculated using weighted t-tests, Wilcoxon rank-sum tests, or Chi-square tests as appropriate. The IPTW-weighted model estimated the average treatment effect after balancing measured baseline covariates | | | | | | | | |
