# Supplementary material for: Inflammatory biomarkers and 30-day thoracic outcomes after surgical versus non-surgical management of spontaneous pneumothorax: a retrospective cohort study
Source: Front Med (Lausanne). 2026 Jul 3;13:1868899. doi: 10.3389/fmed.2026.1868899 (PMC13375800; doi:10.3389/fmed.2026.1868899)
Supplement: Supplementary file 3 [file Table_3.DOCX]

| Supplementary Table S3. Crude and IPTW-weighted associations with 30-day post-discharge thoracic complications in the patient-level sensitivity cohort | | | | |
| --- | --- | --- | --- | --- |
| Variables | Crude OR (95% CI) | P-value | IPTW-Weighted OR (95% CI) | P-value |
| Group |  |  |  |  |
| Non-surgical management | Ref |  | Ref |  |
| VATS | 0.34 (0.17, 0.68) | 0.002 | 0.36 (0.18, 0.71) | 0.004 |
| Sex |  |  |  |  |
| Male | Ref |  |  |  |
| Female | 1.12 (0.42, 2.94) | 0.826 |  |  |
| Smoking |  |  |  |  |
| No | Ref |  |  |  |
| Yes | 1.23 (0.68, 2.22) | 0.494 |  |  |
| Age |  |  |  |  |
| ≤44 | Ref |  |  |  |
| ≥45, ≤ 64 | 0.24 (0.11, 0.51) | <0.001 |  |  |
| ≥65 | 1.41 (0.68, 2.92) | 0.355 |  |  |
| BMI | 0.94 (0.85, 1.05) | 0.275 |  |  |
| Pulmonary Comorbidities |  |  |  |  |
| No | Ref |  |  |  |
| Yes | 1.86 (0.95, 3.64) | 0.069 |  |  |
| Location |  |  |  |  |
| Left | Ref |  |  |  |
| Right | 1.37 (0.76, 2.48) | 0.294 |  |  |
| Pulmonary Bullae |  |  |  |  |
| No | Ref |  |  |  |
| Isolated | 0.48 (0.25, 0.90) | 0.023 |  |  |
| Diffuse | 2.75 (1.09, 6.97) | 0.033 |  |  |
| Pneumothorax Volume | 1.01 (0.99, 1.02) | 0.162 |  |  |
| Post-treatment SII | 1.00 (0.98, 1.02) | 0.723 |  |  |
| Post-treatment PLR | 1.01 (0.99, 1.01) | 0.236 |  |  |
| Post-treatment NLR | 1.01 (0.98, 1.05) | 0.502 |  |  |
| Post-treatment LMR | 1.07 (0.90, 1.28) | 0.420 |  |  |
| Post-treatment WBC | 0.93 (0.84, 1.04) | 0.211 |  |  |
| Post-treatment ALB | 0.98 (0.93, 1.03) | 0.454 |  |  |
| Post-treatment HGB | 0.99 (0.97, 1.01) | 0.109 |  |  |
| Drainage time | 1.13 (1.04, 1.19) | <0.001 |  |  |
| Hospital stay after the index procedure | 1.10 (1.05, 1.16) | <0.001 |  |  |
| Antibiotics |  |  |  |  |
| No | Ref |  |  |  |
| Yes | 2.15 (0.99, 4.66) | 0.305 |  |  |
| OR, Odds Ratio; CI, Confidence interval; IPTW, Inverse Probability of Treatment Weighting; SII, Systemic Immune-inflammation Index; PLR, Platelet-to-Lymphocyte Ratio; NLR, Neutrophil-to-Lymphocyte Ratio; LMR, Lymphocyte-to-Monocyte Ratio; VATS, Video-Assisted Thoracoscopic Surgery; WBC, White Blood Cell count; ALB, Albumin; HGB, Hemoglobin. Note: The crude column presents odds ratios derived from standard logistic regression without weighting. These crude estimates are descriptive and should not be interpreted as mutually adjusted effects. The IPTW-weighted column presents the average treatment effect of VATS after balancing measured baseline covariates. In the IPTW-weighted model, only treatment group was included because baseline covariates had already been balanced by propensity-score weights. Baseline covariates were not re-entered into the weighted model to avoid overadjustment. For continuous variables, odds ratios represent the change in odds per 1-unit increase in the original measurement scale. Reference groups were non-surgical management, male sex, no smoking, age ≤44 years, no pulmonary comorbidities, left-sided pneumothorax, no pulmonary bullae, and no antibiotic use. | | | | |
