# Supplementary material for: Inflammatory biomarkers and 30-day thoracic outcomes after surgical versus non-surgical management of spontaneous pneumothorax: a retrospective cohort study
Source: Front Med (Lausanne). 2026 Jul 3;13:1868899. doi: 10.3389/fmed.2026.1868899 (PMC13375800; doi:10.3389/fmed.2026.1868899)
Supplement: Supplementary file 4 [file Table_4.DOCX]

| Supplementary Table S4. Robust and cluster-robust sensitivity analyses of IPTW-weighted treatment-outcome associations | | | | |
| --- | --- | --- | --- | --- |
| Analysis | Outcome or subgroup | Model / comparison | Effect estimate (95% CI) | P-value |
| Overall sensitivity analysis | 30-day post-discharge thoracic complications | IPTW weighted, robust/sandwich SE | OR = 0.38 (0.21, 0.71) | 0.002 |
| Overall sensitivity analysis | 30-day post-discharge thoracic complications | IPTW weighted, cluster-robust SE by patient ID | OR = 0.38 (0.20, 0.73) | 0.004 |
| Overall sensitivity analysis | Hospital stay after the index procedure | IPTW weighted, robust/sandwich SE | β = 1.91 days (0.34, 3.49) | 0.017 |
| Overall sensitivity analysis | Hospital stay after the index procedure | IPTW weighted, cluster-robust SE by patient ID | β = 1.91 days (0.33, 3.50) | 0.018 |
| Exploratory pulmonary comorbidity stratification | No pulmonary comorbidity | Non-surgical: 39/147 (26.5%); VATS: 12/93 (12.9%) | OR = 0.51 (0.25, 1.05) | 0.067 |
| Exploratory pulmonary comorbidity stratification | Pulmonary comorbidity | Non-surgical: 23/53 (43.4%); VATS: 4/27 (14.8%) | OR = 0.17 (0.04, 0.62) | 0.008 |
| Exploratory pulmonary comorbidity stratification | No pulmonary comorbidity | Hospital stay after the index procedure | β = 1.52 days (-0.04, 3.07) | 0.056 |
| Exploratory pulmonary comorbidity stratification | Pulmonary comorbidity | Hospital stay after the index procedure | β = 3.09 days (-0.96, 7.15) | 0.135 |
| Note: Robust sandwich standard errors were used to account for variance estimation after IPTW weighting. Cluster-robust standard errors were calculated by patient identifier to account for within-patient correlation in the episode-based cohort. The pulmonary comorbidity stratified analyses were exploratory; interaction terms were not statistically significant for 30-day post-discharge thoracic complications (P for interaction = 0.142) or hospital stay after the index procedure (P for interaction = 0.475). Therefore, subgroup results should be interpreted cautiously. OR, odds ratio; CI, confidence interval; IPTW, inverse probability of treatment weighting; SE, standard error; VATS, video-assisted thoracoscopic surgery. | | | | |
