# Supplementary material for: Inflammatory biomarkers and 30-day thoracic outcomes after surgical versus non-surgical management of spontaneous pneumothorax: a retrospective cohort study
Source: Front Med (Lausanne). 2026 Jul 3;13:1868899. doi: 10.3389/fmed.2026.1868899 (PMC13375800; doi:10.3389/fmed.2026.1868899)
Supplement: Supplementary file 5 [file Table_5.DOCX]

| Supplementary Table S5. Crude and IPTW-weighted associations with hospital stay after the index procedure in the patient-level sensitivity cohort | | | | |
| --- | --- | --- | --- | --- |
| Variables | Crude β (95% CI) | P-value | IPTW-Weighted β (95% CI) | P-value |
| Group |  |  |  |  |
| Non-surgical management | Ref |  | Ref |  |
| VATS | 1.32 (-0.47, 3.11) | 0.148 | 1.78 (0.05, 3.52) | 0.044 |
| Sex |  |  |  |  |
| Male | Ref |  |  |  |
| Female | -2.01 (-4.80, 0.95) | 0.182 |  |  |
| Smoking |  |  |  |  |
| No | Ref |  |  |  |
| Yes | 3.21 (1.49, 4.93) | <0.001 |  |  |
| Age |  |  |  |  |
| ≤44 | Ref |  |  |  |
| ≥45, ≤ 64 | 2.57 (0.38, 4.77) | 0.022 |  |  |
| ≥65 | 5.28 (3.37, 7.19) | <0.001 |  |  |
| BMI | 0.02 (-0.28, 0.32) | 0.913 |  |  |
| Pulmonary Comorbidities |  |  |  |  |
| No | Ref |  |  |  |
| Yes | 4.08 (1.97, 6.18) | <0.001 |  |  |
| Location |  |  |  |  |
| Left | Ref |  |  |  |
| Right | 0.70 (-1.07, 2.46) | 0.439 |  |  |
| Pulmonary Bullae |  |  |  |  |
| No | Ref |  |  |  |
| Isolated | 0.07 (-2.32, 2.45) | 0.957 |  |  |
| Diffuse | 2.31 (0.32, 4.03) | 0.023 |  |  |
| Pneumothorax Volume | 0.03 (-0.01, 0.07)* | 0.129 |  |  |
| Post-treatment SII | 0.01 (0.01, 0.02)** | <0.001 |  |  |
| Post-treatment PLR | 0.01 (0.01, 0.02) | <0.001 |  |  |
| Post-treatment NLR | 0.19 (0.07, 0.32) | 0.003 |  |  |
| Post-treatment LMR | -0.45 (-0.98, 0.09) | 0.102 |  |  |
| Post-treatment WBC | 0.12 (-0.19, 0.42) | 0.454 |  |  |
| Post-treatment ALB | -0.35 (-0.50, -0.21) | <0.001 |  |  |
| Post-treatment HGB | -0.09 (-0.13, -0.04) | <0.001 |  |  |
| Drainage time | 1.03 (0.99, 1.07) | <0.001 |  |  |
| Antibiotics |  |  |  |  |
| No | Ref |  |  |  |
| Yes | 9.34 (7.01, 11.67) | <0.001 |  |  |
| β, unstandardized regression coefficient; CI, Confidence Interval; IPTW, Inverse Probability of Treatment Weighting; VATS, Video-Assisted Thoracoscopic Surgery. Note: Outcome variable was hospital stay after the index procedure, measured in days. The crude column presents unweighted associations between each listed variable and hospital stay using standard linear regression. These crude estimates are descriptive and should not be interpreted as mutually adjusted effects. The IPTW-weighted column presents the average treatment effect of VATS after balancing measured baseline covariates. In the IPTW-weighted model, only treatment group was included because baseline covariates had already been balanced by propensity-score weights. Baseline covariates were not re-entered into the weighted model to avoid overadjustment. Regression coefficients represent the change in hospital stay in days. For estimated pneumothorax volume, the coefficient represents the change in hospital stay per 1-unit increase in the recorded volume variable. For inflammatory indices, coefficients represent the change per 1-unit increase. | | | | |
